# Evaluation of cardiovascular risk in a lung cancer screening cohort

**DOI:** 10.1136/thoraxjnl-2018-212812

**Published:** 2019-09-26

**Authors:** Mamta Ruparel, Samantha L Quaife, Jennifer L Dickson, Carolyn Horst, Stephen Burke, Magali Taylor, Asia Ahmed, Penny Shaw, May-Jan Soo, Arjun Nair, Anand Devaraj, Emma Louise O'Dowd, Angshu Bhowmik, Neal Navani, Karen Sennett, Stephen W Duffy, David R Baldwin, Reecha Sofat, Riyaz S Patel, Aroon Hingorani, Sam M Janes

**Affiliations:** 1 Lungs for Living Research Centre, UCL Respiratory, University College London, London, UK; 2 Research Department of Behavioural Science and Health, University College London, London, UK; 3 Department of Radiology, Homerton University Hospital NHS Foundation Trust, London, UK; 4 Department of Radiology, University College London Hospitals NHS Foundation Trust, London, UK; 5 Department of Radiology, Royal Brompton Hospital, London, UK; 6 Respiratory Medicine Unit, David Evans Research Centre, Nottingham University Hospitals NHS Trust, Nottingham, UK; 7 Respiratory Medicine, Homerton University Hospital NHS Foundation Trust, London, UK; 8 Thoracic Department, University College London Hospitals NHS Foundation Trust, London, UK; 9 Killick Street Health Centre, London, UK; 10 Wolfson Institute of Preventive Medicine, Barts and London, London, UK; 11 Institute of Cardiovascular Science, University College London, London, UK

**Keywords:** Lung Cancer

## Abstract

**Introduction:**

Lung cancer screening (LCS) by low-dose computed tomography (LDCT) offers an opportunity to impact both lung cancer and coronary heart disease mortality through detection of coronary artery calcification (CAC). Here, we explore the value of CAC and cardiovascular disease (CVD) risk assessment in LCS participants in the Lung Screen Uptake Trial (LSUT).

**Methods:**

In this cross-sectional study, current and ex-smokers aged 60–75 were invited to a ‘lung health check’. Data collection included a CVD risk assessment enabling estimation of 10 year CVD risk using the QRISK2 score. Participants meeting the required lung cancer risk underwent an ungated, non-contrast LDCT. Descriptive data, bivariate associations and a multivariate analysis of predictors of statin use are presented.

**Results:**

Of 1005 individuals enrolled, 680 were included in the final analysis. 421 (61.9%) had CAC present and in 49 (7.2%), this was heavy. 668 (98%) of participants had a QRISK2≥10% and QRISK2 was positively associated with increasing CAC grade (OR 4.29 (CI 0.93 to 19.88) for QRISK2=10%–20% and 12.29 (CI 2.68 to 56.1) for QRISK2≥20% respectively). Of those who qualified for statin primary prevention (QRISK2≥10%), 56.8% did not report a history of statin use. In the multivariate analysis statin use was associated with age, body mass index and history of hypertension and diabetes.

**Conclusions:**

LCS offers an important opportunity for instituting CVD risk assessment in all LCS participants irrespective of the presence of LDCT-detected CAC. Further studies are needed to determine whether CAC could enhance uptake and adherence to primary preventative strategies.

**Key messages**

**What is the key question?**

- What is the prevalence of coronary artery calcium, cardiovascular risk and statin use as primary prevention against cardiovascular disease among a cohort of individuals undergoing lung cancer screening?

**What is the bottom line?**

- Coronary artery calcification was present in 61.9% of lung cancer screening participants and was positively associated with cardiovascular disease risk; 98% qualified for a statin, though less than half reported using one.

**Why read on?**

- These findings highlight the opportunity for influencing cardiovascular disease outcomes through lung cancer screening and suggest that cardiovascular disease risk assessment should be considered for inclusion in lung cancer screening programmes.

## Introduction

In the National Lung Screening Trial (NLST), annual lung cancer screening (LCS) by low-dose computed tomography (LDCT), improved lung cancer-specific mortality by 20% and all-cause mortality by 6.7% compared with annual chest x-ray.^1^ The benefit in lung cancer-specific mortality has since been confirmed by the Dutch-Belgian Lung Cancer Screening Trial (NELSON)^2^ and the Multicentric Italian Lung Detection (MILD) investigators.^3^ Unsurprisingly, following the reduction in lung cancer death in the LDCT arm in NLST, and given the age and smoking history of the cohort, coronary heart disease (CHD) was responsible for the majority of total deaths in the LDCT arm.^1^ Globally, CHD accounts for the greatest number of deaths annually,^4^ and given both lung cancer and CHD risk are associated with increasing age and smoking history, the LCS population is at disproportionately high risk of CHD-related morbidity and mortality.

In this context, there may be an important opportunity for LCS and LDCT to also help reduce CHD mortality, through simultaneous assessment of cardiovascular disease (CVD) risk, via quantification of coronary calcium, a marker of established atherosclerosis. The coronary artery calcium (CAC) or Agatston score, correlates highly with the volume and burden of plaque seen at post mortem and shows an association with incident CHD risk, such that a score of 1000 has been associated with a 10-fold increased risk of all-cause mortality.^5^ In contrast, a CAC score of 0 is associated with low risk, similar to the background population.^6–10^

While CAC is visible on LDCT, differences do exist compared with dedicated cardiac scanning, in protocols and in particular, the absence of electrocardiography (ECG) gating. Non-gated images are subject to motion artefact and run the risk of inadequately estimating the CAC burden. Nevertheless, several studies have shown that CAC assessments on non-gated LDCT scans are comparable to formal ECG-gated CAC measurements^11–14^ with good agreement between the methods. Direct comparison between these methods has demonstrated underestimation of high CAC in 0%–23.4% of cases,^15^ while overestimation of CAC may also occur. However, these discrepancies may not be of clinical significance if undetected CAC in this group does not translate into cardiovascular events, and the high-risk individuals are appropriately managed. Further studies assessing visual CAC scoring in LCS have shown it to be a reliable predictor of cardiovascular events. Importantly, very low event rates have been reported by non-gated LDCT scans in the CAC=0 group, suggesting there may be a utility for visual CAC reporting in the LCS population, although the definitions of ‘event’ and median follow-up durations of these studies were variable.^16–21^

Quantifying CVD risk through assessment of CAC alone is not recommended,^22^ though a recent consensus document advocates reporting CAC on all non-contrast CT chest scans.^23^ Addition of CAC assessment to the Framingham score can refine risk stratification, which may have a particularly meaningful impact on those in the ‘intermediate’ CVD risk group,^6^ though whether this is also true for individuals eligible for LCS is not known. In the UK, the National Institute for Health and Care Excellence (NICE) guidelines advocate assessment of cardiovascular risk using the QRISK2 algorithm. Those deemed to be at high risk, currently defined as having a 10% or greater risk for events over the next 10 years, irrespective of total cholesterol level, should be offered a statin (atorvastatin 20 mg) as part of a wider discussion around lifestyle interventions to reduce their risk.^24^

In the present study, we aimed to explore the value of coronary calcium and cardiovascular risk in asymptomatic LCS participants who did not report a prior history of CHD. In particular, we aimed to determine (1) the prevalence and extent of coronary calcium in LCS participants using a quantification system considered to be an acceptable alternative to Agatston scoring; (2) participant risk estimates using QRISK2 and how this is distributed across CAC scores and, finally, (3) the prevalence and predictors of statin use among those with both high and low risk estimates and CAC burden.

## Methods

### Study design, participants and setting

This cross-sectional study is nested within the Lung Screen Uptake Trial (LSUT), the methods for which have been described previously.^25^ Briefly, individuals aged between 60 and 75, who had been coded in their primary care health record as current smokers within the past 5–7 years, were invited by their primary care physician for a ‘lung health check’ (LHC) at one of two London hospitals between November 2015 and July 2017. The primary aim of LSUT was to test differences in uptake to LCS between individuals randomly allocated to either ‘standard’ invitation materials or targeted materials, designed to engage socioeconomically deprived smokers. Individuals attending the LHC were invited to participate in the study.

Those meeting the US Preventative Services Task Force (USPSTF) criteria for LCS (ie, ≥30 pack-years and quit ≤15 years ago),^26^ or a lung cancer risk of 1.51% as determined by the Prostate Lung Colorectal Ovarian study (PLCO_m2012_) model^27^ or 2.5% as determined by the Liverpool Lung Project (LLP) model,^28^ were offered a LDCT scan to screen for lung cancer. Participants were excluded from the LDCT if they did not have capacity to give consent, their weight exceeded restrictions for scanner (>200 kg), they were unable to lie flat, had poor physical fitness such that radical treatment would be contraindicated, or had had a CT scan of their chest within the previous 12 months. Participants were given written information on the potential benefits and harms of LCS and following a discussion with the research nurse or clinical trials practitioner, were asked to give informed consent to have an LDCT as part of LCS.

Very brief smoking cessation advice (a standardised intervention from the UK’s National Centre for Smoking Cessation and Training)^29^ was given to all current smokers at the LHC, and participants were also randomised to receive details of their local National Health Service (NHS) smoking cessation service or be proactively referred to the smoking cessation service.

### Data collection

Data were prospectively collected by a study practitioner at the LHC. Self-reported demographics (age, sex, ethnicity, education level, Index of Multiple Deprivation (IMD) score and rank), smoking status and history, cardiovascular and lung cancer risk factors (including all those contained in the QRISK2, PLCO_m2012_ and LLP models), history of CHD and number of general practice (GP) attendances in the past year were recorded. Family history was defined as per the QRISK2 model and was assessed as ‘angina or heart attack in a in a first degree relative aged <60 years old’. Hand-held spirometry, height, weight and blood pressure were also recorded.

### LDCT acquisition

Participants undertook the examination via a 16-channel or higher multidetector, non-ECG-voltage-gated CT without the administration of intravenous contrast. Imaging was performed during suspended maximal inspiration. The lung parenchyma (lung apices to bases) was scanned in its entirety in a single craniocaudal acquisition. The field of view selected as the smallest diameter as measured from widest point of outer rib to outer rib large enough to accommodate the entire lung parenchyma. Thin detector collimation (0.5 mm) was used. Images were reconstructed at 0.5–1.0 mm section thickness using standard soft tissue and lung algorithms. Radiation exposures were as low as possible while maintaining good image quality (median 1.2 mSv, IQR 0.9 mSv, 1.7 mSv). The tube potential and tube current-time product varied according to participant body habitus and were between 80 and 120 kVp and between 20 and 80 mAs, respectively.

### Outcome measures

QRISK2 scores were calculated by ClinRisk Ltd using their QRISK2-2017 Java batch processor and used self-reported history of included risk factors. These are estimated QRISK2 scores as we did not have serum cholesterol values for participants as part of the study, and the batch processor substitutes an age-sex-ethnicity-estimate of cholesterol/ high-density lipoprotein ratio when this is presented as missing. Three patients had missing systolic blood pressure values, hence the batch processor substituted an age-sex-ethnicity estimate of systolic blood pressure for these participants. Self-reported use of statins was recorded.

The LDCT scans were single-read by a team of five radiologists with expertise in thoracic CT reporting and experience ranging from 5 to 28 years. Reports included recording of a visual grading of coronary calcium, which was developed and validated in LCS LDCT examinations by Chiles *et al*.^21^ Here, radiologists performed a simple, overall visual assessment, on a per scan basis taking into account an ‘average’ of all coronary arteries. The grades of none, mild, moderate or heavy used have demonstrated good correlation with formal Agatston scores at cut offs of 0, 1–100, 100–1000 and >1000.

### Sample size and statistical analysis

The sample size of the LSUT cohort was based on the primary behavioural research question and has been described in the published protocol.^25^ However, retrospectively, one might reasonably have posited a priori that around two thirds of our sample would have QRISK2 score greater than 20% and one third less than or equal to 20%. One might also reasonably hypothesise that 15% of those with QRISK2≤20% would have visual CAC grade of moderate to heavy compared with a figure of 25% in those with QRISK2 >20%. For 80% power to observe this difference as significant (5% significance level, two-sided testing), we would require 621 subjects (207 with QRISK2≤20% and 414 with QRISK2>20%). In the event, we had 680 subjects, 204 with QRISK2≤20% and 476 with QRISK2 >20%.

Participants who self-reported a prior history of angina, angioplasty, myocardial infarction and coronary artery bypass grafting were considered to have a prior history of CHD and were excluded from the analysis, as were those without an LDCT or with missing QRISK2 scores. Individuals were categorised by QRISK2 into categories of low (0%–10%), moderate (10%–20%) and high risk (≥20%) of CVD. Descriptive statistics were used to determine the demographic and clinical characteristics of individuals in each QRISK2 category. The distributions of QRISK2 scores by CAC grade and prevalence of CAC by grade in each QRISK2 category were summarised and compared. Associations between QRISK2 score and CAC grade were assessed using χ² and multivariate ordinal logistic regression analyses, and differences in QRISK2 scores between CAC categories were evaluated using the Kruskal-Wallis test, with subsequent pairwise comparisons performed using a posthoc Dunn’s test with a Bonferroni correction for multiple comparisons. Next, the prevalence of self-reported statin use was compared by QRISK2 category and bivariate associations between statin use and various clinical and demographic variables were assessed using χ² analysis. A multivariate logistic regression model was used to assess independence in these associations, adjusting for age, gender, smoking status, history of hypertension, history of diabetes and body mass index (BMI). Variables for the final model were selected on the basis of clinical and statistical significance. So, those variables that were significant in univariable analysis were tested and included in the multivariable model, alongside those variables that were deemed to be a priori confounders and variables that were felt to be of important clinical significance.

Agreement between radiologists was assessed for the 5% of LDCT scans that were second-read as part of the quality assurance process, using the weighted kappa (κ_w_) test with quadratic weights. Missing values were excluded from the analyses (and were present for only one variable, IMD rank). Likelihood ratio testing was used for tests of significance, and p≤0.05 were considered significant. Analyses were carried out using STATA V.14.

## Results

**Figure 1:**
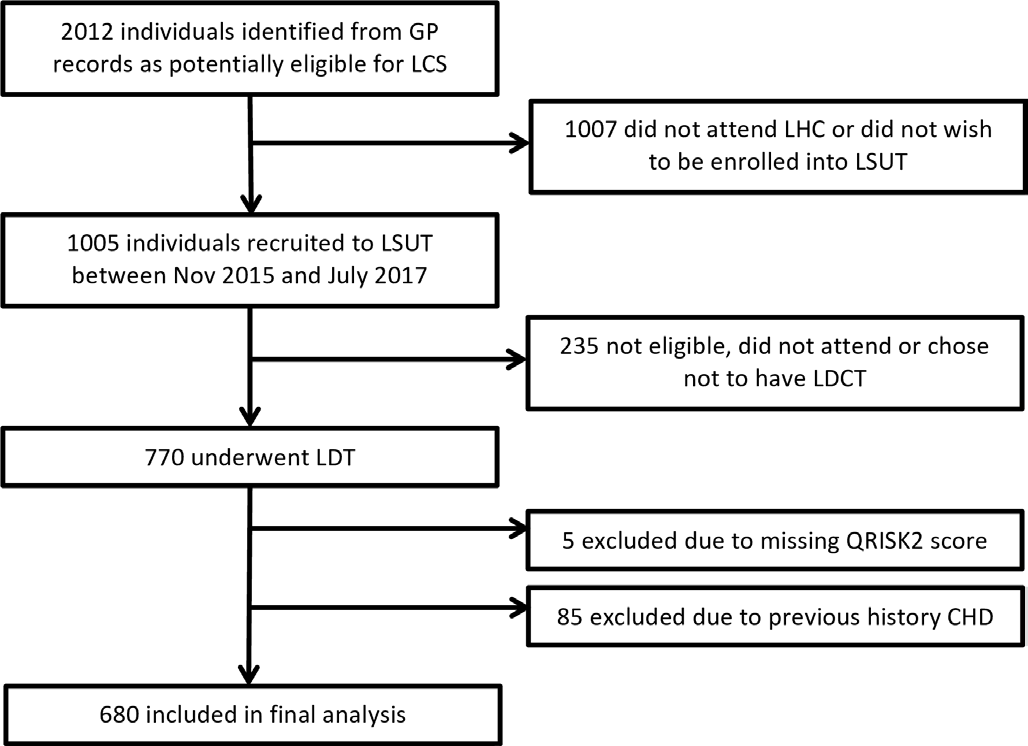
Flow diagram for study participants. CHD, coronary heart disease; LCS, lung cancer screening; LDCT, low-dose computed tomography; LHC, lung health check; LSUT, Lung Screen Uptake Trial.

Out of 2012 potentially eligible individuals identified in the primary care records of 16 GP practices, 1005 attended for an LHC and were recruited into the study. Of those, 770 underwent a baseline LDCT examination. Total 85 participants were excluded due to a history of self-reported CHD and five due to missing QRISK2 score data, leaving a total of 680 participants in the final analysis (figure 1).

**Figure 2:**
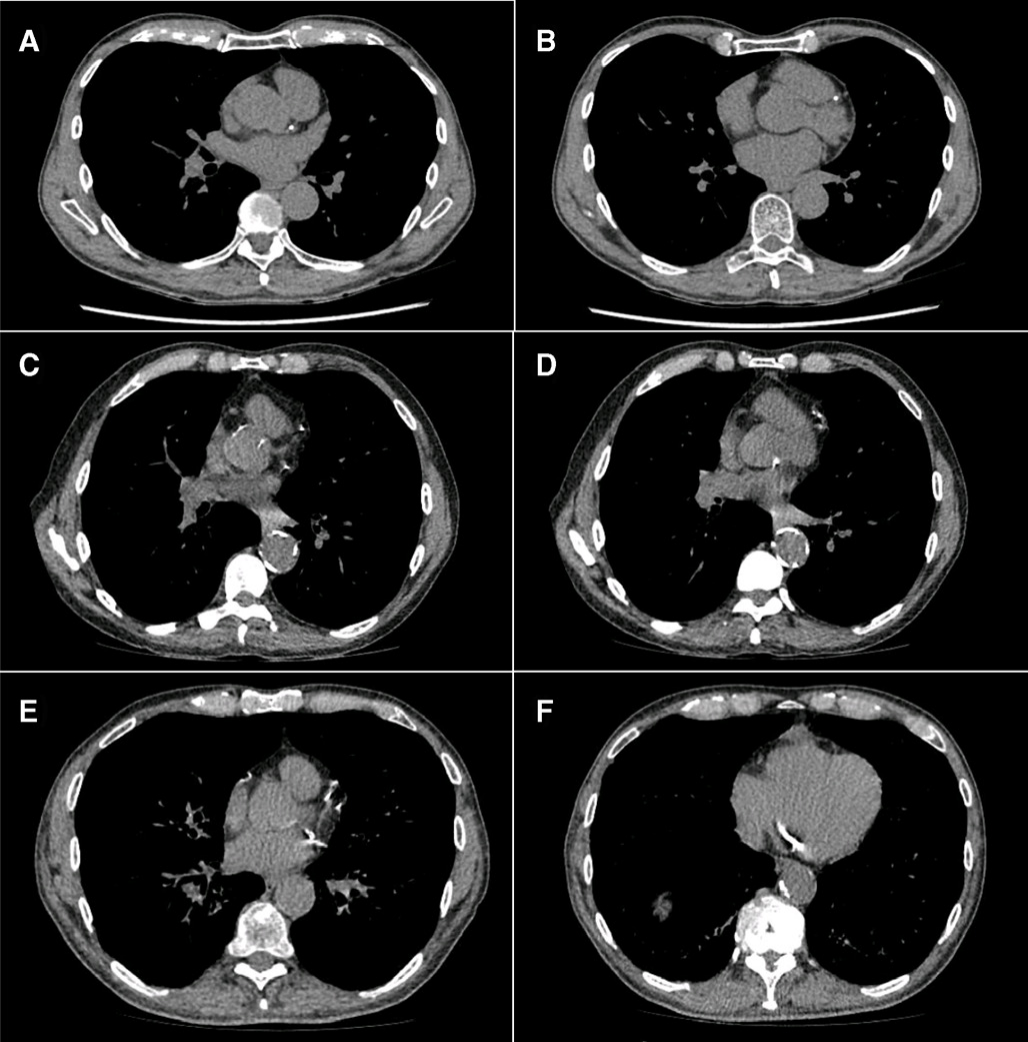
Examples of participants with mild (A,B), moderate (C,D) and heavy (E,F) coronary artery calcification.

Participant characteristics by QRISK2 category are described in table 1 though the low numbers in the lowest QRISK2 group should be considered. 38.4% of the cohort were female (though 12 out of 12 of the QRISK2<10% group were female) and 82.4% were white. More than half of the overall cohort left school at or before the age of 16, though the lower QRISK2 categories tended to be better educated. 88.1% of participants were from the two most socioeconomically deprived quintiles, and this distribution was similar across the three QRISK2 categories. 72.1% of participants were current smokers though as expected, this proportion was higher in the highest QRISK2 category. Smoking intensity and duration and degree of airway obstruction were also higher in the highest QRISK2 category. Similarly, participant BMI and blood pressure also increased by QRISK2 category. Figure 2 shows examples of participants with coronary calcification of mild, moderate and heavy categories.

**Table 1:** Participant characteristics by QRISK2 category (% totals may not sum up due to rounding)

Variables	QRISK2 Score category: n(%) or median (IQR)*			Total
	<10%, n=12	10%–20%, n=192	>20%, n=476	
Age	62 (61, 63)	63 (62, 66)	67 (64, 70)	65 (63, 69)
Female (vs male)	12 (100)	71 (37.0)	178 (37.4)	261 (38.4)
Ethnicity				
White	11 (91.7)	137 (71.4)	412 (86.6)	560 (82.4)
Black (African or Caribbean)	0 (0)	32 (16.7)	41 (8.6)	73 (10.7)
Other	1 (8.3)	23 (12.0)	23 (4.4)	47 (6.9)
Highest level of education				
Left school at or before age 15	5 (41.7)	76 (39.6)	276 (58.0)	357 (52.5)
CSEs, O-levels or equivalent	2 (16.7)	22 (11.5)	41 (8.6)	65 (9.6)
A-levels or equivalent	1 (8.3)	24 (12.5)	42 (8.8)	67 (9.9)
Further education	0 (0)	14 (7.3)	19 (4.0)	33 (4.9)
Bachelor degree	3 (25.0)	26 (13.5)	52 (10.9)	81 (11.9)
Further higher degree	1 (8.3)	23 (12.0)	40 (8.4)	64 (9.4)
IMD quintile				
1 (most deprived)	5 (41.7)	88 (45.8)	276 (58.0)	369 (54.3)
2	5 (41.7)	78 (40.62)	147 (30.1)	230 (33.8)
3	0 (0)	3 (1.56)	13 (2.7)	16 (2.4)
4	0 (0)	0 (0)	1 (0.2)	1 (0.2)
5 (least deprived)	0 (0)	0 (0)	0 (0)	0 (0)
Smoking status				
Current smoker (vs former)	2 (16.7)	112 (58.4)	376 (79.0)	490 (72.1)
Years smoked (years)	45 (42–27)	45 (43, 48)	49 (45, 52)	47 (44, 51)
Average smoking intensity (cigs/day)	10 (7–18)	15 (10, 20)	20 (12, 20)	20 (10, 20)
Lung function				
FEV1 (% predicted)	89 (78.5, 106)	84 (69, 100)	81 (63, 94)	81 (66, 96)
FEV/FVC (%)	66 (62, 77)	70 (65, 76)	67 (60, 74)	68 (61, 75)
Other cardiovascular risk factors				
On hypertensive treatment	0 (0)	34 (17.7)	196 (41.2)	230 (33.8)
Family history of heart disease	1 (8.3)	54 (28.12)	233 (49.0)	288 (42.4)
History of diabetes	0 (0)	0 (0)	83 (17.4)	83 (12.2)
BMI (kg/m^2^)	22.9 (19.8, 30.2)	25.5 (22.1, 28.6)	26.1 (23.1–29.3)	25.9 (22.8, 29.2)
Systolic BP (mm Hg)	114 (100, 121)	129 (117, 138)	139 (128, 152)	135 (125, 148)
Diastolic BP (mm Hg)	73 (70, 75)	82 (75, 87)	84 (77, 91)	83 (76, 90)

BMI, body mass index; BP, blood pressure; IMD, Index of Multiple Deprivation.

Out of the 680 participants, 421 (61.9%) had CAC present on LDCT. CAC grade was ‘moderate’ in 145 (21.3%) and ‘heavy’ in 49 (7.2%) (table 2). CAC grade was associated with QRISK2 category (p<0.01). Overall 98% of participants had a QRISK2 score of ≥10% and were therefore eligible for statin primary prevention. Conversely, more than half (54.7%) of the participants with a QRISK2 of 10%–20% had no CAC visible.

**Table 2:** Prevalence of CAC on LDCT by QRISK2 score category

QRISK2 risk category	Visual CAC grade, n(%)					P value
	None	Mild	Moderate	Heavy	Total	
<10%	10 (83.3)	2 (16.7)	0 (0)	0 (0)	12 (1.8)	<0.01
10%–20%	105 (54.7)	58 (30.2)	24 (12.5)	5 (2.6)	192 (28.2)	
>20%	144 (30.3)	167 (35.1)	121 (25.4)	44 (9.2)	476 (70.0)	

CAC, coronary artery calcification; LDCT, low-dose computed tomography.

**Figure 3:**
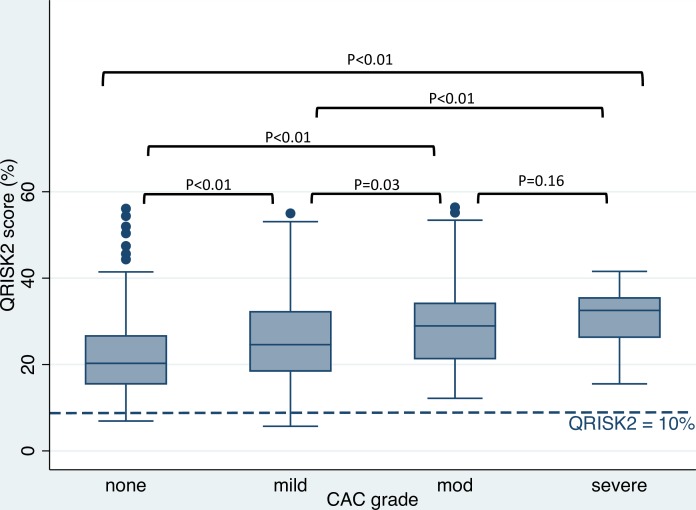
QRISK2 score distribution for each visually determined CAC grade on LDCT. The boxes contain the 25th to 75th QRISK2 scores within each category, with the median value represented by a solid line running through the box. The whiskers extend to the upper and lower adjacent values and the dots represent the outliers. An extra horizontal dotted line has been added to demonstrate the threshold for initiation of statin therapy for primary prevention in the UK (10%). Posthoc p values corrected for multiple comparisons between group medians are highlighted. CAC, coronary artery calcification; LDCT, low-dose computed tomography.

The range of QRISK2 scores within each CAC group was wide. Median QRISK2 scores increased significantly with increasing CAC grade, H(3) = 70.428, p<0.01. Adjusted p values for multiple pairwise comparisons of median QRISK2 scores between CAC groups were all significant except between the ‘moderate’ and ‘heavy’ CAC grades (figure 3). This association was supported by increasing ORs for each increasing QRISK2 category (table 3).

**Table 3:** Unadjusted ordinal logistic regression for association between LDCT CAC grade and QRISK2 category

QRISK2 category	OR (CI) (unadjusted)	P value
<10%	1.0	
10%–20%	4.29 (0.93–19.88)	0.062
≥20%	12.29 (2.69–56.1)	<0.01

CAC, coronary artery calcification; LDCT, low-dose computed tomography.

Self-reported statin use was associated with QRISK2 (p<0.01) and number of GP visits in the past year in the univariate analysis (table 4). Of those that did qualify for a statin based on their QRISK2, 56.8% did not report a history of statin use, with this number being even higher (76.6%) in the 10%–20% QRISK2 category. 90% of participants who were not taking statins reported visiting their GP ≥1 times in the past year. In the multivariate analysis, statin use was independently associated with age, history of hypertension, diabetes and BMI (but not number of GP visits in the past year) (table 5). Several variables including gender, IMD score and quintile, education level, smoking status, years smoked, years quit, pack-years and systolic blood pressure were not found to be associated with statin use in the univariate analysis or after adjusting for other variables. Gender and smoking status were included in the final model as they were felt to be important a priori confounders with regards to development of CAC.

**Table 4:** Number of individuals qualifying for a statin based on QRISK2 alone compared with self-reported history of statin use, and with self-reported number of GP attendances in the past year

	On statin	Not on statin	P value	Not on statin, when indicated by QRISK2 score (%)
QRISK2 score category, n (% of row)				
<10%	0 (0)	12 (100)	<0.01	0
10%–20%	45 (23.4)	147 (76.6)		76.6
>20%	237 (49.8)	239 (50.2)		50.2
Total	282	386		56.8
Number of GP attendances in past year, n (% of column)				
0	10 (3.6)	47 (11.8)	<0.01	
1–5	211 (74.8)	285 (71.8)		
>5	61 (21.6)	66 (16.4)		

**Table 5:** Univariable and multivariable logistic regression model for history of self-reported statin use

	OR (CI) (unadjusted)	P value	OR (CI) (adjusted)	P value
Age				
60–63	1	<0.01	1	<0.01
64–67	1.29 (0.88 to 1.90)		1.51 (0.98 to 2.35)	
68–71	2.08 (1.36 to 3.18)		2.36 (1.45 to 3.84)	
72–76	2.20 (1.32 to 3.66)		2.42 (1.35 to 4.31)	
Gender				
Male	1	0.27	1	0.12
Female	1.19 (0.87 to 1.61)		1.31 (0.93 to 1.87)	
Smoking status				
Former	1	0.11	1	0.14
Current	0.76 (0.54 to 1.06)		0.75 (0.51 to 1.10)	
History of hypertension				
No	1	<0.01	1	<0.01
Yes	4.26 (3.03 to 5.96)		3.51 (2.44 to 5.06)	
History of diabetes				
No	1	<0.01	1	<0.01
Yes	13.55 (6.85 to 26.8)		11.10 (5.44 to 22.6)	
BMI				
<18.5	0.91 (0.27 to 3.02)	<0.01	1.65 (0.47 to 5.80)	0.023
18.5–25	1		1	
>25	1.64 (1.14 to 2.36)		1.64 (1.14 to 2.36)	

BMI, body mass index.

Inter-observer agreement for the double-read LDCT scans was very good according to Landis & Koch^30^ (κw=0.88, p=0<0.01).

## Discussion

In this prospective observational study in a cohort of individuals undergoing an LDCT examination for LCS, we have found that 62% of participants have coronary calcium present. Second, increasing QRISK2 was associated with increasing CAC grade on LDCT. Third, 98% of LCS-eligible individuals met the ≥10% 10-year CVD risk threshold required for statin primary prevention of CVD events in the UK, although less than half reported a history of statin use. In the adjusted analyses, statin use was associated with factors related to increasing cardiovascular risk, but not with frequency of prior GP visits within the past year. These data add to the debate on the role LDCT could play for enhancing parallel CVD prevention efforts as part of wider LCS programmes nationally and personalising risk management strategies.

Our data first confirm previous reports that LCS-eligible individuals have evidence of coronary disease.^18 21^ A recent study reporting 10-year outcomes in a subcohort of participants who met the USPSTF criteria^26^ for LCS from the Multi-Ethnic Study of Atherosclerosis (MESA) noted an almost threefold increase in the cardiovascular event rate (20.8%) in this group^31^ than the 10-year event rate reported in the overall MESA cohort (7.8%).^32^ This finding highlights the potentially sizeable impact implementation of primary prevention in LCS-eligible individuals could have.

Our findings of a positive association between CAC grade and QRISK2 are in keeping with prior studies.^33^ Ultimately, whether CAC grade offers any clinical utility over and above the QRISK2 score for risk prediction remains a key question as most patients by virtue of age and smoking would be considered at high risk. In the context of LDCT, it is of note that a large number (54.7%) of participants in the moderate QRISK2 category (10%–20%) had no CAC. It has been suggested from the LCS cohorts that only a small number will have a CVD event.^16–21^ However, many of these studies only looked at fatal CHD events or had limited follow-up duration. More recent data from the USPSTF-eligible cohort in MESA discussed above^31^ demonstrated the 10-year CVD event rate (including fatal and non fatal coronary events or stroke) to be 14.2% in this group and may be explained by the presence of non-calcific atherosclerotic disease. The wide range of QRISK2 scores in those with no CAC in the present study also supports the notion that this group cannot be assumed to have a benign outcome, though the fact that women or certain ethnic groups may have clinical risk overestimated should also be considered. Increasingly, data are emerging that combining CVD risk scoring with clinical risk factors plus CAC may offer a much more refined approach to risk stratification with greater net reclassification, especially of individuals in the intermediate risk categories.^6 34^ However, in the context of LDCT, outcome studies with CAC would be needed to demonstrate the added value of risk scoring using CAC versus QRISK2 alone.

Nonetheless, 98% of LCS participants were at greater than 10% risk by QRISK2, the threshold currently recommended in the UK at which statin therapy can be considered. This proportion may be higher than expected, based on results from other LCS screening studies; however, those cohorts have typically been slightly younger and have included fewer current smokers.^1 35–37^ In another recent report of another ‘real-world’ UK-based CT screening pilot, a similarly high QRISK2 scores were observed.^38^ Despite this, our analysis found that only approximately 50% who qualified, reported statin use, falling to 23% in the 10%–20% QRISK2 category. This result is consistent with other UK and US studies, who have reported 46.0% and 49.7% statin use in their eligible cohorts, respectively.^39^ In the UK, individuals aged between 40 and 74 are invited to NHS health checks in order to carry out CVD risk assessment, though uptake to this has been low at only 30% in 2012.^40^

Overall, these findings support the use of LDCT and LCS as another opportunity to engage high-risk patients for primary prevention of CVD. While it remains uncertain if CAC can improve risk stratification over and above standard risk estimation, reporting CAC using the visual grading method used in the present study is quick and may be motivational in discussions with patients and for initiating and adherence to statin and antihypertensive therapy. Certainly a systematic review has demonstrated that CAC screening improved medication adherence and could likely motivate further behavioural changes.^41^ A large body of research^42^ implicates patient-related, behavioural barriers in non-adherence to statins (eg, concerns about side effects, misconceptions about causality and symptoms, low perceived benefit) and has shown that behavioural interventions targeting these barriers are effective.^43^ In the UK NHS health checks, initiation of statin treatment only occurs in 20% of NHS health check attendees with ≥20% CVD risk.^40^ Awareness of the presence of CAC may, therefore, have a positive effect on statin use in this group, particularly if barriers among those who previously declined treatment could be identified and addressed. Furthermore, as novel preventative therapies emerge, the uniformly high CVD risk in this group (65.7% with ≥20% 10 year risk) may warrant more discerning risk stratification strategies for which knowledge of CAC may be informative.

The current study has some limitations. First, CAC was graded visually, and not using formal Agatston scoring, however, this is an accepted and validated method^21^ and interobserver agreement in this study for 5% of scans that were double read was very good. The lack of cardiovascular event data limited our ability to examine if CAC adds any additional clinical utility to standard risk scores. Larger planned studies with longer follow-up will address this in due course. The inclusion criteria in the present study targeted socioeconomically deprived smokers aged 60–75,^25^ but we feel results are still generalisable, given emerging evidence advocating selection of LCS-eligible individuals based on lung cancer risk.^44 45^ For screening populations including younger participants, it should be considered that the distribution of cardiovascular risk may be lower than that observed in this study. We did not collect data on NHS health check attendance; on reasons for the lack of statin use if prescribed or if discussions had taken place about statins at all. Finally, we did not measure serum cholesterol and so our QRISK2 score used a substitute value as well as self-reported history of risk factors, which may make the scores less accurate. Measuring serum cholesterol may not add more value given the high risk of participants due to other (smoking and age) risk factors and adds expense and time. Despite these limitations, our study was carried out in a practical and pragmatic manner with high uptake rates, making it generalisable to a population and real world setting.

## Conclusion

Patients undergoing LDCT, as part of an LCS programme, have a high prevalence of coronary disease estimated through visual scoring of CAC and are also at high CVD risk according to standard clinical calculators. However, despite this, a substantial proportion of patients are not on statin therapy for primary prevention, despite eligibility based on 10 year CVD risk scores. We propose that LCS programmes may offer a unique opportunity to engage patients for CVD prevention by (1) helping address the underutilisation of statins in this high risk group and (2) potentially using anatomical evidence of CAC as motivation to enhance adherence to preventative interventions.
